# The Efficacy of Pre-Operative Self-Isolation Guidelines for Safe Elective Orthopaedic Surgeries: A Prospective Pilot Study

**DOI:** 10.7759/cureus.27280

**Published:** 2022-07-26

**Authors:** Ashwin Bhadresha, Chiranjit De, Nachappa Sivanesan Uthraraj, Vusumuzi Sibanda, Kalsoom Altaf, Leonidas Mitrogiannis, Jai Relwani

**Affiliations:** 1 Trauma and Orthopaedics, William Harvey Hospital, Ashford, GBR

**Keywords:** pcr screening, covid-19, elective orthopaedic surgery, self-isolation guidance, compliance

## Abstract

Objectives

This study aimed to determine the efficacy of the self-isolation guidance for elective orthopaedic surgery. We aimed to evaluate the relationship between patient compliance with the self-isolation guidance and the resulting COVID-19 status. This would give planning strategies for managing elective orthopaedic lists.

Method

For the study, 110 patients who underwent elective orthopaedic surgical procedures during a one-month period were identified. Patients scheduled for surgery were asked to take a SARS-CoV-2 PCR test three days prior to surgery and they were asked to follow the self-isolation guidance. On the day of admission, patients declared compliance with self-isolation regulations. Admission was refused in cases of non-compliance. After discharge, telephone calls were made to patients to determine the degree of compliance with the self-isolation guidance.

Results

Overall, 106 out of 107 patients that were compliant with the self-isolation guidance tested negative for COVID-19; 15 patients had their operation cancelled over the one-month period; of which one-third were cancelled by the patients themselves. Three patients were found to be non-compliant with the self-isolation guidance. Of these three non-compliant patients, one tested positive for COVID-19. Adherence to the self-isolation guidelines helped to prevent last-minute cancellations and manage the list effectively.

Conclusions

Compliance with our self-isolation guidance accompanied by PCR screening minimises the risk of testing positive for COVID-19 and is thus an effective system to safely perform elective orthopaedic surgery. Intentionally overbooking theatre lists by 10 to 12.5% may account for cancellations and improve theatre efficiencies during post-pandemic recovery plans for elective orthopaedic surgeries.

## Introduction

The global outbreak of coronavirus disease caused by SARS-CoV-2 (COVID-19) has contributed to over 340 million infections globally and more than 5 million deaths in a short time frame [1,2]. The scale of the pandemic has created significant pressures on healthcare systems globally, resulting in the cessation of non-urgent elective surgery to free up beds and medical staff for treating patients with COVID-19 [3-5].

However, in order to prevent the colossal pressures mounting on the healthcare system, National Health Service England has adopted safe measures to resume elective services in a safe COVID-free environment through green pathways [6-8]. Although no universal guidelines exist, there are several local guidelines in place in different hospitals. The common principal actions to achieve this include SARS-CoV-2 vaccination, self-isolation with polymerase chain reaction (PCR) testing before admission, adherence to practices that reduce the risk of community-acquired SARS-CoV-2 infection such as social distancing and shielding, and hospital staff screening [9-11].

Our hospital conducted a polymerase chain reaction (PCR) test three days before surgery followed by self-isolation for three days. If the test was positive for an infection, surgery was postponed until this was clear.

The aim of this study was to determine the efficacy of the self-isolation guidance for elective orthopaedic surgery. We evaluated the patient compliance with the pre-operative self-isolation guidance, the efficacy of guidance, and the resulting positivity rate of COVID-19 after either being compliant or non-compliant with self-isolation guidelines. We aimed to ascertain if non-compliance with the COVID-19 isolation guidance resulted in the cancellation of the operation or contraction of COVID-19 infection.

## Materials and methods

This single-centre prospective cross-sectional cohort study included 110 patients who underwent elective orthopaedic surgical procedures during a four-week period in the month of October 2021 at The Elective Orthopaedic Centre at Canterbury; which is a designated cold site developed following the green pathway guidelines. By the 31st of October, there were 17,368 cases of COVID-19 in Canterbury. The sample size of 110 patients was deemed sufficient for the scope of this study and for the study aims because we have collected data on the entire population undergoing surgery at the Elective Orthopaedic Centre in Canterbury during the month of October. We did not have any control over the sample size because we worked within a fixed time frame and this was a pilot study. All elective orthopaedic surgery data were extracted from the digital medical record system in line with local clinical governance protocols and we obtained IRB approval. The elective surgeries in this study included upper and lower limb arthroplasties, arthroscopic surgeries, non-urgent elective spine surgeries, and other elective orthopaedic operations for joints, peripheral nerves, tendons, and bones. Patients who underwent emergency or urgent surgeries were excluded from this study. Although there may have been slight discrepancies in exposure to COVID-19 with these varying procedures, over a third of the operations were day cases, with 80% of patients having an inpatient stay of less than 72 hours. Therefore, these patients would have been subjected to a similar level of COVID-19 exposure. Furthermore, the majority of these patients would have had either a general or spinal anaesthetic with sedation. Therefore this would have also standardised the COVID-19 risk to a certain degree. 

Patients scheduled for elective surgery took a SARS-CoV-2 PCR test three days before the surgery and were asked to follow the self-isolation guidance for these three days prior to their surgery. The duration of three days was chosen because this was in accordance with the local trust guidelines at East Kent. We are aware that the self-isolation guidance for green elective pathways can differ to a certain degree in varying trusts, but in our trust patients were required to take a PCR test three days prior to surgery and were asked to follow the self-isolation guidelines. Table 1 summarises the locally followed guidelines for self-isolation and the patients were interviewed through the same questionnaire for this prospective study. If the test showed a positive result, surgery was postponed until the patient and their household was clear of the infection. On the day of admission, the patients declared that they have followed the self-isolation guidance and had not had any symptoms or contact with COVID-19 patients. Although clinical assessment in itself does not rule out being positive or negative for COVID-19, these patients were negative three days prior to surgery on a PCR test, and if adherent to the self-isolation guidelines, there will be a low false negative rate. Admission was refused in all cases of non-compliance with any of the set protocols.

Once the patient had been discharged from the hospital, we made telephone calls with a four-month follow-up to the patients to determine the degree of compliance with the self-isolation guidance, and to ascertain if any patient contracted COVID-19 during the post-operative period. In order to minimize inter-observer bias, all the questions asked to the patients by the authors in this study were standardised and strictly followed by the locally implemented guidelines for self-selection as outlined in Table 1.

**Table 1:** Standardised and locally implemented pre-operative self-isolation guidelines.

We will ask you and your household to self-isolate for 3 days before surgery. This means you need to:	
1	Stay at home.
2	Not go to work, school, or public areas.
3	Not attend any gatherings. This includes gatherings of friends and families in private spaces, for example, family homes, weddings, and religious services
4	Avoid having visitors to your home.
5	Ask friends, family members, or delivery services to carry out errands for you – such as getting groceries, medications, or other shopping.
6	Strictly avoid contact with someone who is displaying any symptoms of coronavirus (COVID-19). These symptoms include high temperature, a new and continuous cough, or loss of taste or smell.

## Results

The demographic profile of the study population is summarized in Table 2. During the study period, a total of 120 patients were listed to contact via telephone to answer the compliance questionnaire. However, 10 patients opted not to participate with the questionnaire and were thus excluded from the study. Therefore, in total, we acquired data on 110 patients who underwent elective orthopaedic surgery.

**Table 2:** Demographic profile of the study population.

Total patients (N)	110
Age (years)	
mean	63.2
SD	13.5
Gender Distribution	
Male	45 (40.9%)
Female	65 (59.1%)
ASA grade	
ASA-1	42 (38%)
ASA-2	41 (37%)
ASA-3	26 (24%)
ASA-4	1 (0.9%)
ASA-5	0
Smoking status	
Smokers	92 (83.6%)
Non-smokers	18 (16.3%)

Table 3 shows the distribution of different surgical procedures with regard to the orthopaedic sub-speciality. Additionally, it also lists the number of cancellations among the different sub-specialities. The cancellations had no particular pattern or statistical significance with respect to the different sub-specialities. Therefore, it is not possible to draw a succinct conclusion as to which sub-specialities are more akin to face cancellations. This study primarily aims to reflect overall on elective orthopaedic surgeries.

**Table 3:** Distribution of the elective orthopaedic surgeries and the number of cancellations with respect to different orthopaedic sub-specialities.

Sub-specialty	Total surgeries performed	No of Cancellations
Shoulder	13 (11.8%)	3
Elbow	3 (2.7%)	1
Hands	5 (4.5%)	0
Hips	32 (29%)	3
Knees	36 (32.7%)	4
Foot and Ankle	11 (10%)	3
Spine	10 (9%)	1

Table 4 shows the length of hospital stay of the patients. Most of the patients were operated on as day cases (36.3%). However, there was no statistically significant difference (p=0.31) among the different sub-groups in terms of hospital stays. Therefore, the final outcome has no variation with regard to the hospital stay of the patients.

**Table 4:** Length of hospital stay of the patients.

Length of Stay (N=110)	
Day Case	40 (36.3%)
24hr - <48hr	32 (29%)
48hr - <72hr	17 (15.4%)
>72hrs	21 (19%)

Table 5 shows the number of cancellations within each respective sub-speciality. There were 15 cancellations in total in our study cohort. Table 5 conveys if the reason for the cancellation was hospital- or patient-dependent. Patient cancellations only include situations where the patient cancelled of their own volition. This included if they had COVID-19, personal circumstances and if they deemed themselves too unfit for surgery at the time. Hospital cancellations include not adhering to the pre-operative isolation guidance, contracting COVID-19, illness prior to surgery, and being medically unfit for surgery. Of the 15 cancellations, two patients were due to the patient testing positive for COVID-19 test results. One out of these two patients was cancelled due to the patient cancelling on their own accord, and the other one was due to cancellation by the hospital. However, these cancellation patterns and the COVID-19 test results were not statistically significant with a p-value of 0.643 (>0.05).

**Table 5:** Cancellation status and COVID-19 test results of the patients.

Cancellation Status	COVID-19 Test Results		P-Value
	Negative	Positive	
Cancelled by hospital	10	1	0.643
Cancelled by patient	5	1	

Table 6 compares the patient compliance with the self-isolation guidance to the COVID-19 status. This demonstrates that for the two patients who tested positive for COVID-19, one patient was compliant with the self-isolation questionnaire. However, one patient did not comply with the self-isolation guidance. Of the 108 patients who tested negative for COVID-19, 106 were compliant with the self-isolation guidance, and only two patients did not follow the guidance. Importantly, COVID-19 test results and compliance with the set guidelines were statistically significant with a p-value of 0.001 to 3 decimal points (<0.05).

**Table 6:** Compliance with the isolation guidelines and COVID-19 test results of the study population.

COVID-19 Test Result	Compliant to guidelines		P-Value
	Yes	No	
Positive	1	1	0.000034
Negative	106	2	

## Discussion

To our knowledge, this study is the first to describe the compliance of elective orthopaedic patients with the pre-operative self-isolation guidance to ascertain its efficacy. The significant findings of this study are twofold. Firstly, 106 out of 107 patients that were compliant with the self-isolation guidance tested negative for COVID-19. Furthermore, in patients who are non-compliant with the pre-operative guidance, there has been one in three chances of subsequently testing positive for COVID-19 with a statistically significant association. This, therefore, demonstrates that strong compliance with the self-isolation guidance will likely result in patients not going on to develop COVID-19. Secondly, of the total of 120 patients listed for elective orthopaedic surgery in our centre in October 2021, there were a total of 15 cancellations. In order to maximise theatre efficiencies, the authors of this study propose that overbooking theatre by approximately 10 to 12.5% on a monthly basis will account for the lost theatre output caused by cancellations. However, this would require preparing a pool of patients who are available on short notice and happy to accept the fact that their surgery might be deferred on close notice as well This is purely a hypothesis at this point and will need data involving a higher number of patients across different centres for proven efficacy.

A comparable study by Nishitani et al. evaluated the acceptability of the 14 days of self-quarantine and the positivity rate of pre-operative SARS-CoV-2 PCR screening for patients undergoing elective orthopaedic surgery in Japan. They found that of all the patients who completed the self-isolation quarantine programme, no patient tested positive for COVID-19 [3]. The findings of this study support our study and the validity of the self-isolation guidance. In Japan, patients were asked to self-quarantine for 14 days followed by PCR testing upon admission. However, in our study protocol, patients took a PCR test three days before surgery followed by compliance with the self-isolation guidance. This represents the negligible differences in pre-operative COVID-19 guidelines between different countries and healthcare systems. However, both the studies achieved similar outcomes. There is no consensus on repeating the PCR test for screening purposes. Adamson et al. reported only a 1.9% rate of positive PCR after initially negative PCR results and support selective repeat PCR testing only when symptoms develop after a negative test, or in hospitalized patients with a high clinical suspicion for COVID-19 [12]. Besnier et al. have tried providing some novel ideas to reinstate elective surgery. However, this study was done amidst the pandemic and therefore the results would vary in the current scenario [13]. Other studies from different parts of the world have tried to reinforce the safe resumption of elective orthopaedic surgery [14-16]. However, different health care systems are facing different challenges. This study has tried to address one of the main aspects of elective surgeries during the current time in the form of pre-operative self-isolation guidelines.

Strict compliance with our self-isolation guidance with PCR screening three days prior to surgery is beneficial in decreasing the possibility of COVID-19 in patients scheduled to undergo elective orthopaedic surgery. As mentioned in our methods section, the duration of three days was chosen because this was in accordance with our trust guidelines at East Kent. In our study cohort of 110 patients, there was only one patient who tested positive for COVID-19 after following the self-isolation guidance. This finding is supported by a recent study by Kader et al. which calculated that in the United Kingdom, the risk of patients with an undetected SARS-CoV-2 infection being inadvertently admitted for elective orthopaedic surgery was found to be as low as 0.07% which is 1 in 1400 [17]. An international multicentre observational study by COVIDSurg Collaborative reported a 30-day mortality of 19.1% in elective surgical patients with a peri-operative infection of COVID-19 [18]. Importantly, in this cohort of elective patients, pulmonary complications accounted for 53.1% of deaths. The other causes of death included haemorrhagic, infectious, and other medical and surgical-specific complications. No deaths were attributable to undetected COVID-19 infection. This, therefore, stresses the utmost importance of minimising pre-operative transmission of COVID-19. As a result of the extrapolation of this fact, compliance with our self-isolation guidance plays a crucial role in decreasing the mortality rate amongst elective orthopaedic patients.

In this study, we found that there was a total of 15 cancellations over the month; 10 were hospital-dependent, and five were patient-dependent. This is a significantly high number of cancellations (12.5%), and the lead author proposes that intentionally overbooking theatre lists at our trust can be one of several strategies adopted to reduce the overall rate of case cancellations in order to maximise theatre efficiencies. The burden of cancelled operations during the pandemic has also been noted elsewhere in the literature. Chiu et al. reported a cancellation prevalence of 8% for elective orthopaedic surgery at a hospital in Hong Kong. They suggest the implementation of an integrated pre-operative preparation system in order to significantly decrease the rate of same-day intended surgery case cancellations [19]. Furthermore, COVIDSurg Collaborative found that 81.7% of operations for benign conditions would be cancelled due to the 12 weeks of peak disruption due to COVID-19 [20]. This, therefore, emphasises the need to implement recovery plans and strategies to restore surgical activity safely.

One of the limitations of our study was that our self-isolation guidance requires voluntary cooperation from patients. Furthermore, our study method of actively telephoning patients by asking about their level of compliance with each question of the self-isolation guidance also relies on their duty of candour. We believe that the majority of our patients would have adequately complied with the self-isolation guidance and also would have been honest in relaying their response to the study since we explicitly mentioned to every patient the anonymity of the study protocol. This study did not include a control group, and thus a control group without self-quarantine and without a PCR test three days prior to surgery may be useful to scientifically determine the efficacy of our programme. However, in the current study, we aimed to do a risk assessment and therefore it was not crucial to have a control group to determine the compliance with our self-isolation programme. Furthermore, in the current scenario, it is not a safe practice to have a control group for elective surgery due to the increased risk of bringing COVID-19 into the hospital. We also acknowledge that multicentre studies involving a higher number of patients would provide more evidence on the isolation guidelines. However, this is one of the primary studies which has tried to develop a holistic approach for pre-operative isolation guidelines for elective orthopaedic surgeries.

## Conclusions

In conclusion, this study showed that compliance with our self-isolation guidance accompanied by PCR screening minimises the risk of testing positive for COVID-19 and is an effective system to safely perform elective orthopaedic surgery. Intentionally overbooking theatre lists by 10-12.5% may account for cancellations and improve theatre efficiencies during post-pandemic recovery plans for elective orthopaedic surgery.
